# Working Hours Associated with the Quality of Nursing Care, Missed Nursing Care, and Nursing Practice Environment in China: A Multicenter Cross-Sectional Study

**DOI:** 10.1155/2023/8863759

**Published:** 2023-11-06

**Authors:** Miqi Li, Ying Wang, Meichen Du, Hui Wang, Yanqun Liu, Brianna N. Richardson, Jinbing Bai

**Affiliations:** ^1^Tongji Hospital, Tongji Medical College, Huazhong University of Science and Tecnology, Wuhan, Hubei, China; ^2^Center for Women and Children Health and Metabolism Research, Wuhan University School of Nursing, Wuhan University, Wuhan, Hubei, China; ^3^Nell Hodgson Woodruff School of Nursing, Emory University, Atlanta, GA, USA

## Abstract

**Aim:**

The aim of the study was to examine the effect of the length of working hours on missed nursing care, quality of nursing care, and perceptions of the nursing practice environment.

**Methods:**

A multicenter cross-sectional investigation using online questionnaires was conducted from April 2 to May 10, 2022, in twenty nine hospitals (13 Level-III hospitals and 16 Level-II hospitals). We collected data on the working hours of nurses and nurse-reported outcomes, including missed nursing care, quality of nursing care, and nursing practice environment. Restricted cubic spline (RCS) regression models were used to examine relationships between the hours per shift and nurse-reported outcomes.

**Results:**

We investigated 12,703 nurses with a response rate of 97.33%. Nurses worked on average 7.72 (SD = 1.16) hours on the day shift and 8.92 (SD = 2.20) hours on the night shift, respectively. On the day shift, working 7.5 hours shift showed minimal missed nursing care; meanwhile, working 7–7.5 hours were correlated with the highest satisfaction of the nursing practice environment and better quality of nursing care. On the night shift, the highest missed nursing care was found for a working duration of 12 hours to the working 7 hours, with the lowest satisfaction while better quality was observed. The percentage of nurses who reported working overtime was 30.33%. Nurses who worked overtime reported lower satisfaction and poorer quality of nursing care on all shifts; moreover, working overtime showed the positive correlation to missed nursing care on the day shift, while on night shift was not statistically significant.

**Conclusion:**

Positive outcomes were observed for nurses who reported working 7–7.75 hours on the day shift and 12 hours (no more than 15 hours) on the night shift. *Implications of Nursing Management*. The results reemphasized the need for managers to reduce the working hours, overtime work, and the frequency of the night shift.

## 1. Introduction

Although the working hours for nurses predominately fall between 36 and 40 hours weekly, the reality of the number of hours nurses work, especially per shift, is still up for debate. Prevailing shift patterns can be categorized into morning, day, evening, and night depending on what time the work started[1]. Generally, the day shift starts at 6 am or 7 am and ends before 9 pm, whereas the evening or night shift frequently lasts from 6 pm to 2 am or 11 pm to 7 am [2, 3].

On the other hand, shift patterns are usually classified into quickly forward rotating shift and slowly rotating shift based on the shift length. The rapidly rotating shift with approximately 8 hours per shift time is usually adopted in China [4] and Korea [5]. It is associated with a shorter duration (<12 hours) of about 4, 8, or 10 hours. Meanwhile, the slowly rotating shift is longer, commonly over 12 hours and even up to 24 hours. In the UK, US, and several European Union countries, longer shifts have become a controversial topic, specifically ones that last a consecutive 12 hours [3].

Many research studies investigated the correlation between work shift or the length of working hours of nurses and nurses' or patients' outcomes. Recent studies have actively examined the effect of different shift lengths on nurse-reported outcomes and found that long working hours per shift can shorten the handover times and reduce the overlap to provide continuous patient care [6, 7]. Some nurses preferred working long shifts for the benefits, including better work-life balance and more time off [1].

However, inappropriate working hours had a harmful impact on nurse, hospital, and patient outcomes. For the nurse, previous studies showed a strong positive relationship between long daily working hours and adverse nurse outcomes, including fatigue [8], burnout [9], lapses of attention, sleepiness [10], and increased rates of sick leave [11]. For the hospital, excessive working hours often lead to an overly stressful work environment, which was a reason that nurses reported leaving their jobs [9]. In turn, high turnover rates further exacerbated the shortage of nurses and increased overtime work. Experience from Magnet hospitals demonstrated that improving the practice environment could reduce job dissatisfaction, burnout, or intention to leave [12].

Furthermore, for the patient, insufficient breaks for nurses were considered as a factor that was harmful to patients' outcomes. Much evidence supported that overtime work was positively correlated with missed nursing care (MNC), which caused mediation errors and increased patients' fall with injury [13, 14]. A study reported that all shifts >8 hours were associated with increasing rates of MNC [15]. In addition, nurses working 12 hours or longer was positively associated with poor quality of nursing care, high errors rates, and poor patient safety [16]. D'Sa et al. conducted a 2-year longitudinal study and found that excessive monthly working hours was related to increased patients' infection rates as well as higher mortality rates [17].

To maximize the benefits of shift nursing work, managers and researchers developed different work schedules. In Korea, a survey of 312 nurses reported working the day shift for an average of 8.87 hours and the night shift for 10.57 hours [18] and 33.3% of the nurses (out of 2,568 nurses) worked more than 12 hours per shift in the UK. In contrast, nurses in Cambodia were on call for 24 hours and worked normal 8-hour shifts [19]. In the US, the government policy limited nurses' hours to less than 12 hours per day [20]. A large cross-sectional survey indicated that most day shifts were 8 hours in European countries [15]. The discussion of the night shift referenced circadian rhythm with 8, 10, and 12 hours being acceptable durations for work. Current studies also indicated that the long shifts (+12 hours) and the short shifts were less common [1]. From the nurses' opinion, the 10- and 12-hour night shifts were sustainable to balance juggling children, families, and work schedules with their partners.

In general, clinical managers introduced mixed patterns with nurses alternating between working a long and then a short shift [21]. There were many studies that investigated the relationship between mixed shifts and their outcomes, showing that mixed patterns led to a higher cost and resource use and made it easier to ignore patients' needs [15, 22]. Nonetheless, the outcomes of different working hours in diverse shift, especially by day and night shifts, combined with the circadian rhythm are ambiguous. Although previous studies and practices have documented advantages of different nurse work schedules; however, they were limited to small sample sizes and the lack of recommended length of work, and more evidence is needed to consider both work scheduling and working hours.

We conducted a multicenter cross-sectional study to describe Chinese nurses' work schedule characteristics. We discussed the impact of the length of different shifts on nurses' perception of the nursing practice environment (NPE), MNC, and quality of nursing care (QNC). Furthermore, we recommended beneficial working hours based on day and night shifts. We intend to provide a reference for managers' scheduling and improve quality of nursing care. For policymakers, this study provided great foundation for the physical and mental health of nurses that can be used to develop work schedules with fair working hours.

## 2. Materials and Methods

### 2.1. Design and Sample

We conducted a hospital-based, multicenter, cross-sectional investigation in Hubei province, China, from April 2 to May 10, 2022. Registered nurses were investigated in this study. At one Level-III hospital and one Level-II hospital, we utilized random cluster sampling from each prefecture-level city in Hubei province. According to China's hospital grading system, hospitals were classified into three levels from Grade III to Grade I and Grade III hospitals represent the advanced medical level and nursing competence [23]. Since 3 cities did not have Level-III hospitals, a total of 29 hospitals were enrolled, including 13 Level-III hospitals and 16 Level-II hospitals. Nurses were included if they were (a) registered nurses working in an inpatient department unit and (b) willing to participate in the study. Nurses who were not on duty due to vacation, sick leave, or training were excluded. The researchers submitted the questionnaire and informed consent online which would create a link. The participants filled out questionnaires via the online link. Questionnaires and instructions for data collection were emailed to the nursing directors of each hospital to distribute and conduct the survey.

### 2.2. Independent Variable

#### 2.2.1. Working Hours

Working hours were measured based on nurses' self-report. Nurses were asked about scheduling, scheduled working hours, actual working hours, and overtime based on their last shift. Overtime was defined as work time in which nurses exceeded the scheduled working hours specified for their shift.

### 2.3. Dependent Variables

#### 2.3.1. Quality of Nursing Care

Nurses reported the quality of care in their unit as “poor,” “average,” “good,” or “excellent” during their last shift. As demonstrated in earlier studies, the quality of nursing care by nurses' self-report was reliable and valid [24–26].

#### 2.3.2. Missed Nursing Care

MNC was considered an error of omission, in which nursing activities prescribed for the patient were either partially or completely omitted or significantly delayed. It was measured using the Chinese version of the Missed Nursing Care Scale (MNCS) developed by Kalisch and Williams [27] and translated by Si [28]. The scale included two parts. Part A describes 24 nursing activities rated based on the frequency of them being missed as follows: (0) “never,” (1) “rarely,” (2) “occasionally,” (3) “frequently,” and (4) “always.” Total scores of the subscale part A range from 0 to 96, with higher scores indicating higher levels of MNC. Part B uses 19 items to investigate the causes with 4 dimensions as follows: management, communication, labor resources, and material resources. The reasons nursing care was missed were expressed as (3) “significant reason,” (2) “moderate reason,” (1) “minor reason,” and (0) “no reason.” Total scores of subscale part B ranged from 0 to 57. The higher the score, the more it proved to be the main reason for missed nursing care. The content validity with a S-CVI 0.93, a Cronbach's *α* of 0.924 in part A, and a S-CVI of 0.98, a Cronbach's *α* of 0.916 in part B. The range of factor loading was between 0.487 and 0.855 and explained 64.308% of ANOVA.

#### 2.3.3. Nursing Practice Environment

In the current study, the NPE was measured by the Practice Environment Scale-Revised, which contains 10 dimensions, 36 items, and 1 overall evaluation item [29]. Each item was assigned a value of 0–100 points, with “0” indicating very dissatisfied or strongly disagree and “100” indicating very satisfied or strongly agree. That means the total scores of this scale range from 0 to 3700, with higher scores indicating a better practice environment. The NPE had a good construct validity with a RMSEA of 0.043, a GFI of 0.943, and a AGFI of 0.930. The overall Cronbach's *α* coefficient of the scale was 0.983, and each dimension's Cronbach's *α* ranges from 0.846 to 0.94.

### 2.4. Control Variables

The included covariates were based on the nursing staff demographic characteristics, complexities, severity, and nursing grades. The following control variables were considered as follows: (1) nursing staff demographic characteristics: age, gender, education level, working seniority (<2 years, 2–5 years, 5–10 years, 10–20 years, and >20 years), and professional and technical title (nurse, senior nurse, nurse in charge, assistant director nurse, and above) and (2) department characteristics: (a) working department included internal medicine, surgery, gynecology and obstetrics, pediatric, neonatology, others inpatient department, intensive care unit (ICU), neonatal intensive care unit (NICU), pediatric intensive care unit (PICU), and specialist intensive care unit (specialist ICU). (b) The nurse-to-patient ratio (N-P ratio) refers to the number of patients reported by nurse in the last shift. (c) The patient-care grade ratio refers to the number of patients with different patient-care grades in the last shift. The patient-care grade was divided into special-level nursing, first-level nursing, second-level nursing, and third-level nursing, which classified based on the health industry standard WS/T431-2013 of China considered patient's condition and the Barthel Index. The higher the degree of dependence and the more severe the disease, the higher the patient-care grades, so the highest grade was special-level nursing and the lowest grade was third-level nursing [30]. (d) Nurse's overtime work situation (yes/no).

### 2.5. Statistical Analysis

Data were imported from the network platform into SPSS for analysis. In descriptive statistics, frequency and percentage were used for enumeration data. Numerical variables were described using the mean and standard deviation (SD) or median and upper and lower quartiles when the data did not follow a normal distribution. Differences between groups were tested by one-way ANOVA, Kruskal–Wallis/Wilcoxon rank sum test, Kendall tau-b correlation coefficient, and simple linear regression. All the priori levels of significance and hypothesis tests were 2 sided.

Candidate variables were carefully chosen based on clinically relevant and significant univariate relationship factors. On univariate analysis, variables with a *p* value <0.25 were selected and entered a multivariable model. In the current study, the generalized linear models such as logistic regression and multiple regression were difficult to describe the nonlinear relationship between the independent and dependent variables. Restricted cubic spline (RCS) are popular way to flexibly model nonlinear relationships in regression models. RCS is based on the cubic spline which use cubic polynomials to obtain a smooth function, which add beyond boundary knots (before the first and after the last) to improving the behavior at the extremes [31]. Therefore, we conducted the RCS model to examine the optimum length of work in MNC, quality of nursing care, and nurse practice environment perception. In the models, all the categorical variables were classified into dummy variables and special secondary school, nurse, <2 years, and internal medicine ward were as the reference variables of the education level, profession-technical title, work seniority, and department, respectively.

We selected different cutoff points for working hours, according to the advice of Harrell in regression modeling strategies [32, 33]. We used the RCS with three knots at the 10th, 50th, and 90th (0.10, 0.50, and 0.90), four knots at the 5th, 35th, 65th, and 95th (0.05, 0.35, 0.65, and 0.95), five knots at the 5th, 27.5th, 72.5th, and 95th (0.05, 0.275, 0.50, 0.725, and 0.95), and six knots at the 5th, 23th, 41th, 59th, 77th, and 95th (0.05, 0.23, 0.41, 0.59, 0.77, 0.95) to flexibly model the association of working hours, MNC, QNC, and NPE. The nonlinear associations were conducted using the Chi-square test. We calculated the ORs for quality associated with work hour. A two-sided *p* value <0.05 was considered significant. Statistical analyses were performed using IBM SPSS Statistics 23.0 and R software.

### 2.6. Ethical Considerations

The study was approved by the Ethics Committee of Tongji Hospital, Tongji Medical College, Huazhong University of Science and Technology (no. TJ-IRB20220454).

## 3. Results

### 3.1. General Characteristics of the Participants

In total, 13,052 of the nurses completed the survey and 349 questionnaires were excluded due to uncompleted answers. Finally, 97.33% questionnaire datasets of the study containing 12,703 nurses were included in analysis. On average, nurses worked 7.72 (SD = 1.16) hours and took care of 7(P_25_–P_75_, 4–10) patients on the day shift; 12.85%, 41.03%, 65.71%, and 28.57% patients with special-level care, first-level care, second-level care, and third-level care, respectively. On the night shift, work hours were 8.92 (SD = 2.20) hours and per nurse should took care of 13 (P_25_–P_75_, 6–25) patients, which in 19.99% of special-level care, 23.33% of first-level care, 38.46% of second-level care, and 21.05% of third-level care. 82.47% nurses come from Level-III hospitals and 97.2% of them were female. The majority were from internal medicine (32.8%), with a bachelor's degree (84%). 46.6% of the nurses held a senior professional-technical title and the mean age was 32.07 years (SD = 6.79). Employment of 5–10 years was reported the most in terms of work seniority. During the nurses' last shift, 69.5% was on duty during the day and 30.33% nurses reported working overtime (Table 1).

### 3.2. Prevalence of MNC, NPE, and QNC

The most frequently MNC was changing sheets. The most significant causes of MNC were labor resources, management, communication, and material resources. 43.9% of the nurses rated their unit's QNC as “good.” The average score of NPE was 3081.88 (SD = 739.37).

### 3.3. Association between RNs' Working Hours and MNC, NPE, and QNC

All the data on the RCS analysis were divided by shift (day and night). The variables of age, gender, overtime work, and patient-care grade ratio were significant in univariate analysis and showed a bias. We found that when the age, gender, overtime work, and patient-care grade ratio were not fed into the RCS models of night shift of MNC, NPE, and day shift of QNC, the RCS models had a good performance.

**Figure 1:**
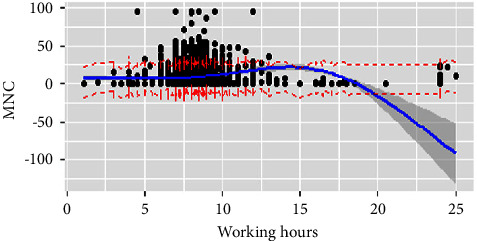
Working hours and MNC on the day shift. Figure legend: Relationship between working hours and missed nurse care on the day shift using a restricted cubic spline line regression model. The missed nurse care score is depicted by the black dot and ratios are indicated by solid lines and 95% CIs by shaded areas. The working hours at six knots of 5th, 23th, 41th, 59th, 77th, and 95th are 7 hours, 7.5 hours, 8 hours, 8 hours, 8.2 hours, and 9.25 hours.

**Figure 2:**
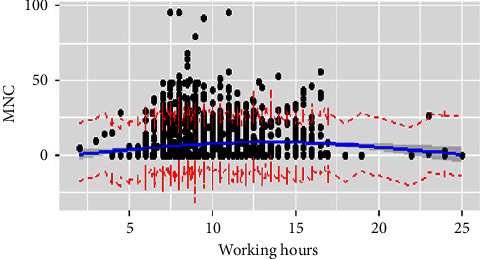
Working hours and MNC on the night shift. Figure legend: Relationship between working hours and missed nurse care on the night shift using a restricted cubic spline line regression model. The missed nurse care score is indicated by the black dot and ratios are indicated by solid lines and 95% CIs by shaded areas. The working hours at three knots of 10th, 50th, and 90th are 7 hours, 8.5 hours, and 12 hours.

We conducted RCS to investigate the association between nurses' working hours and MNC (Tables2 and 3). The RCS with 6 knots and 3 knots were prioritized for the interpretation of the effect of working hours on MNC for the day and night shift, respectively. On the day shift, working hours, overtime work, education, professional-technical title, department, labor resources, and management were significantly correlated with MNC and explained 10.3% of the variation. We visualized the relationship in Figure 1. The shape of the curve showed a slight negative association when the duration was less than 7.5 hours and increased visibly from 7.5 hours to 15 hours, indicating a significant positive relationship. The curve presented a sharp drop, which revealed a negative correlation for working beyond 15 hours. In other words, working up to 7.5 hours showed minimal MNC but was greatest for shifts exceeding 15 hours. Overtime work, nurses' postgraduate education background, the professional-technical title of nurse in charge and assistant director nurse or above, labor resources, and management showed a positive relationship with MNC. The gynecology and obstetrics unit and NICU were negatively associated with MNC. For the night shift, working hours, professional-technical title (assistant director nurse or above), departments (surgery, pediatric, and specialist ICU), patient-care grades (special-level care and second-level care), labor resources, communication, and management explained the 9.7% variance in the model. Except departments which were negatively associated, the other independent variables showed a positive correlation with MNC. With a tipping point of 12 hours, the length of work was positively related to MNC when it was less than 12 hours. Conversely, it showed a negative relation. That is, nurses reported the highest MNC when they worked 12 hours on the night shift (Figure 2).

**Figure 3:**
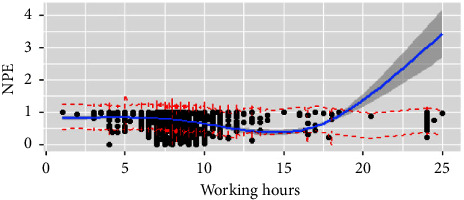
Working hours and NPE on the day shift. Figure legend: Relationship between working hours and nurses' practice environment satisfaction on the day shift using a restricted cubic spline line regression model. The missed nurse care score is shown by the black dot and ratios are indicated by solid lines and 95% CIs by shaded areas. The working hours at four knots of 5th, 35th, 65th, and 95th are 7 hours, 7.75 hours, 8 hours, and 9.25 hours.

**Figure 4:**
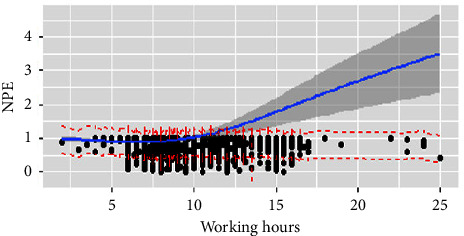
Working hours and NPE on the night shift. Figure legend: Relationship between working hours and nurse's practice environment satisfaction on the night shift using a restricted cubic spline line regression model. The missed nurse care score is shown by the black dot and ratios are indicated by solid lines and 95% CIs by shaded areas. The working hours at four knots of 5th, 35th, 65th, and 95th are 7 hours, 7.75 hours, 8 hours, and 9.25 hours.

Tables 2 and 4 and Figures 3 and 4 describe the correlation between working hours and perceptions of NPE. For the day shift, overtime work, nurse-to-patient ratio, and special-level nursing were negatively correlated with NPE satisfaction. Meanwhile the surgery unit, others unit, NICU, PICU, specialist ICU, and first-level nursing had a positive relation. In terms of the night shift, work experience of 5–10 years and second-level nursing were negatively associated with NPE satisfaction; however, the surgery unit, NICU, and specialist ICU were positive. The significant variables explain 5.9% and 6% of NPE satisfaction on the day and night shift, respectively. The satisfaction on the day shift remained stable below 7 hours of working time but goes down at 15 hours (Figure 3). Nevertheless, on the night shift, the satisfaction increased gradually and followed an unnoticeable decline at 7–7.75 hours (Figure 4). Essentially, we observed the highest NPE satisfaction when nurses worked 7–7.5 hours on the day shift and the lowest was reported when nurses worked 7 hours on night shift.

The number of working hours as compared to QNC is shown in Tables 5 and 6. On the day shift, the findings revealed a significantly lower OR which trended to the poor quality of nursing care as follows: overtime working (OR 0.5 (95% CI 0.45–0.55)), working seniority of 2–5 years (OR 0.79 (95% CI 0.76–1.35)), 5–10 years (OR 0.74 (95% CI 0.60–0.90)), 10–20 years (OR 0.77 (95% CI 0.62–0.95)), >20 years (OR 0.65 (95% CI 0.51–0.84)), and nurse-to-patient ratio (OR 0.99 (95% CI 0.99-0.99)). The other units (OR 1.25 (95% CI 1.10–1.42)), NICU (OR 1.44 (95% CI 1.05–2.01)), and specialist ICU (OR 1.42 (95% CI 1.10–1.84)) suggested better quality of nursing care. On the night shift, senior nurse (OR 0.04 (95% CI 0.00–0.27)) and 2–5 years (OR 0.79 (95% CI 0.62–0.99)) showed poorer quality of nursing care; yet, the OB-GNY unit (OR 1.36 (95% CI 1.04–1.79)), pediatric (OR 1.37 (95% CI 1.01–1.85)), second-level nursing (OR 1 (95% CI 1.00-1.00)), and third-level nursing (OR 1 (95% CI 1.00-1.00)) were observed as having better quality of nursing care.

## 4. Discussion

The results found that about one third of the Chinese nurses worked overtime, an average of 7.72 hours on the day shift and 8.92 on the night shift. In 2018, a survey of 1449 nurses in Guangdong province, China, noted that 55% of the nurses reported working overtime [34]. However, we would like to attract attention to the way that nurses working overtime have evolved into a common phenomenon all over the world. A cohort study showed that 50.5% of the Denmark nurses were scheduled for long shifts (≥9 h–<12 h), 1–12 times in the past year [3]. Also, for Finland were 46.6% and averaged between 8.2 and 8.4 hours [35]. In Malaysia, most nurses reported working extended hours. At least 50% of the nurses had experienced doing a double shift and about 30% worked during their day off [36]. In the midwestern United States, 97.7% of the NICU nurses worked greater than 10 hours [37]. The overtime observed in this investigation is better compared to those studies previously mentioned. This improvement may be due to the emphasis placed by the Chinese and Hubei provincial governments on health workers.

An agreement was reached that overtime was negatively correlated with the quality of nursing care and nurses' health whether or not it was mandatory or voluntary overtime [38]. Therefore, in 2014, the American Nurses Association advocated for nurse working time to be limited to 12 hours or less per day [39]. As the Chinese government focused more attention on nurse welfare and construction of nurse staff [40] and the Hubei provincial governments' concern for nurses' mental and physical health [41], the number of nurses working overtime decreased significantly. However, the length of work time for the day/night shift that was conducive to quality of nursing care was undefined, especially in Chinese samples. Thus, in this study, we explored the appropriate duration for the day/night shift to improve patient safety.

The results of MNC showed that 7–7.5 hours on the day shift and less than 12 hours on the night shift reported the minimum missed of nursing care. When it comes to 15 hours on the day shift, MNC were more likely to be reported. This was consistent with previous studies which found that longer shifts were prone to missed care [16, 22]. It was probably related to the fact that the time setting was circadian rhythm [42]. In China, for the nurses working a full-time job in the hospital, it is more acceptable for nurses worked <8 hours on the day shift or 12 hours on the night shift. The nurses in our study may have preferred that night shifts not exceed 12 hours. The results of a survey of Wuhan, Hubei province, showed that 40.6% of the night nurses were willing to participate in public activities such as training during their breaks [43], it is because that almost hospital in Hubei province have set the “sleepy day” (it is on the day after night shift and only for sleeping) that nurses can get enough rest.

Moreover, a strong positive correlation can be seen in that overtime work led to a tripling incidence of missed care in both shifts. The results presumably associated to the insufficient nurse staffing. Although the nurse-to-patient ratio was not significant in the RCS model, the proportion of the patient-care grade and reason of labor resource which nurse reported was conspicuously to MNC. The results were in line with previous studies [44], which perceived staffing adequacy for nurse with a greater impact on missed care than the actual nurse-to-patient ratio. Regarding nurse characteristics, our study showed that nurses who held a postgraduate education degree and higher professional-technical title were more likely to have MNC. Our explanation for these results was that both kinds of nurses may take more indirect nursing work such as management and scientific research. Bragadóttir's et al.'s study corresponded with our study in that age and role (practice nurse or retested nurse) were significantly related to MNC [45]. However, at present, two systematical reviews indicated that there was not a consistent influence or effect of nurses' characteristics on MNC [46–48]. Thus, to affirm these findings, future research will require a large sample, more rigorous design, and expansive scope.

A good practice environment was important for talent engagement and retention, especially influencing the quality of nursing care and patient safety [49].

In this study, we investigated working hours and its impact on nurses' evaluation of the practice environment. Day staff reporting a better work environment than night staff was supported by previous studies [50]. Nurses gave the highest rating to the practice environment when they worked 7–7.5 hours and lowest when they worked more than 15 hours on the day shift. On the one hand, the appropriate working hours reflected a more comprehensive hospital management and nursing workflow. On the other hand, the avoidance of fatigue allowed nurses to have a more positive work experience [37], which is consistent with a study in Malaysia. Nurses working extended hours had more negative perceptions to the practice environment. In addition, nurses who were not working during their day off had a more positive association with the practice environment [51]. On night shift, nurses who worked 7 hours reported the most negative perceptions of the practice environment. The probable reason may be that the shorter the length of night shift work, the more frequent the night shift work for nurse. Plenty of studies have indicated that abnormal emotions were usually showed up on nurses who were on duty of continual night shifts [37, 52]. Shift work may function as an occupational stressor that impacts nurses' perceptions of the practice environment and increase nurses' intention to leave [53].

The present study found that appropriate work time benefited the quality of nursing care. Similar to NPE, the working hours for the day and night shift were recommended to be 7–7.5 and 15 hours, respectively. Day staff showed better quality of nursing care than night staff, possibly due to nurses' sustained attention and predicted cognitive efficiency under long shifts [10]. To short shift, nurses were preferred on the day shift. In regards to long shifts, there is substantial evidence between the working hours and quality of nursing care. The long shift was harmful to nurses' sleep which led to lower quality of nursing care [54]. However, studies showed that the long shift contributed to the continuous patient care [21]. In contrast, it has been reported that there is no statistically significant difference in the quality of nursing care and shift length in a recent study [55].

Although evidence showed that schedule night shift of no longer than 8 hours [56], long shift (over 8 hours but no more than 15 hours) on night shift was suggested in this study, particularly combined with the high nurse-to-patient ratio. It was noted that nurse staffing was an important factor to the quality of nursing care. In the general inpatient units, nurses usually cared for more patients on the night shift than the day shift, whereas nursing work of the night shift was less than day time. In another words, patient-care hours was increased under a long shift [21]. In intensive care units, all the patients were with high dependence, so they were rated a higher patient-care grade. There was not much difference of nursing work between the day and night shifts. Therefore, the short shift in the ICU is still to be considered for the impact of nurse staffing and the patient-care grade on the quality of nursing care.

## 5. Conclusion

This study identified a better work duration using the perspective of nurse-reported outcomes. Working hours of 7–7.75 on the day shift and 12 hours (no more than 15 hours) on the night shift were recommended from the comprehensive of MNC, nurse-reported quality of nursing care, and perceptions of NPE. This study also highlighted the diverse roles of units, important positive impact of less overtime, and better work experience (included the work seniority and professional-technical title) in the outcomes. However, the mechanisms are still under discussion. Thus, there is an urgent need for researchers to explore how a long duration of overtime work or rotation impacts the quality of nursing care. However, despite the strength of our large sample size and representation of the study, there were several limitations. We surveyed nurses' schedules who only reviewed one shift. Studies have provided that continuous shift working/overtime working was different from occasional shift working/overworking [57]. In addition, although we controlled for department units as a confounding variable, we did not delve into the relationship between work time and nursing outcome under the difference units.

## Figures and Tables

**Table 1:** Characteristics of the study participants (*N* = 12703).

Characteristics		*N* (%)	Mean (SD)	Range
Hospital grade	Level-III	10476 (82.47)		
	Level-II	2227 (17.53)		

Departments	Internal medicine	4166 (32.80)		
	Surgery	3629 (28.57)		
	Gynecology and obstetrics	854 (6.72)		
	Pediatric	660 (5.20)		
	Neonatology	59 (0.46)		
	Others	1761 (13.86)		
	ICU	728 (5.73)		
	NICU	274 (2.15)		
	PICU	81 (0.64)		
	Specialist ICU	491 (3.87)		

Age (years)			32.07 (6.79)	20∼60
≤25		1830 (14.41)		
26–34		7473 (58.83)		
35–44		2565 (20.19)		
≥45		835 (6.57)		

Gender	Male	361 (2.84)		
	Female	12342 (97.16)		

Educational background	Special secondary school	77 (0.61)		
	Junior college	1836 (14.45)		
	Undergraduate	10669 (83.99)		
	Postgraduate	121 (0.95)		

Overtime work	Yes	3853 (30.33)		
	No	8850 (69.67)		

Shift	Day	8831 (69.52)		
	Night	3872 (30.48)		

Work seniority (years)	<2	1372 (10.81)		
	2–5	2008 (15.80)		
	5–10	4088 (32.18)		

Work seniority (years)	10–20	3938 (31.00)		
	>20	1297 (10.21)		

Professional-technical title	Nurse	2066 (16.26)		
	Senior nurse	5917 (46.58)		
	Nurse in charge	4304 (33.88)		
	Assistant director nurse or above	416 (3.28)		

Missed nursing care (top3)^a^				0∼4
Changing sheets		5627 (7.03)		
Emotional support to patient and/or family		5182 (6.47)		
Turning patient every 2 hours		4846 (6.05)		

Causes of missed nursing care^b^				0∼18
Management		62740 (25.92)	4.94 (5.26)	
Communication		62464 (25.81)	4.92 (5.16)	
Labor resources		82455 (34.07)	6.49 (3.90)	
Material resources		34379 (14.2)	2.71 (2.81)	

Quality of nursing care	Poor	22 (0.17)		0∼4
	Average	1317 (10.37)		
	Good	5577 (43.90)		
	Excellent	5787 (45.56)		

Satisfaction of the nursing practice environment			3081.88 (739.37)	0∼3700

*Note*. ^a^: *N* = rarely(1) + occasionally(2) + frequently(3) + always(4). ^b^: *N* = significant reason(3) + moderate reason(2) + minor reason(1).

**Table 2:** Comparison of MNC/NPE across groups (*N* = 12703).

Characteristics	MNC			NPE		
	Mean (SD)/Media (P_25_, P_75_)	*F*/*t*/*Z*	*p*	Mean (SD)/Media (P_25_, P_75_)	*F*/*t*/*Z*	*p*
N-P ratio^1^		3.411	0.001		−4.749	<0.001
Departments^2^		6.03	0.001		7.41	0.001
Internal medicine	6.83 ± 10.58			3030.97 ± 759.15		
Surgery	6.35 ± 10.40			3112.28 ± 722.00		
Gynecology and obstetrics	4.65 ± 8.91			3118.84 ± 752.76		
Pediatric	6.07 ± 9.89			3011.01 ± 767.28		
Neonatology	2.78 ± 5.62			3150.34 ± 688.13		
Others	6.29 ± 11.37			3105.76 ± 735.90		
ICU	6.44 ± 10.29			3030.54 ± 754.73		
NICU	4.24 ± 8.29			3206.7 ± 666.497		
PICU	3.99 ± 9.09			3292.48 ± 660.604		
Specialist ICU	6.49 ± 10.60			3197.8 ± 644.661		
Age (years)^3^		10.688	0.014		36.522	<0.001
≤25	1 (0, 8)			3290.5 (2666.5, 3673)		
26–34	1 (0, 9)			3380 (2720, 3686)		
35–44	1 (0, 9)			3420 (2824, 3682)		
≥45	1 (0, 11)			3239 (2646.5, 3635.5)		
Gender^2^		1.114	0.265		−2.820	0.005
Male	6.91 ± 11.273			3297 (2506.5, 3681.5)		
Female	6.29 ± 10.410			3372 (2727.75, 3681)		
Education background^3^		13.80	0.003		81.881	<0.001
Special secondary school	0 (0, 8)			3281 (2850, 3649.5)		
Junior college	1 (0, 8)			3190 (2504.25, 3632)		
Undergraduate	1 (0, 9)			3394 (2755, 3686)		
Postgraduate	3 (0, 16)			3475 (2960.5, 3676)		
Overtime work^2^		−25.913	<0.001		−22.178	<0.001
Yes	4, (0, 16)			3099 (2382, 3594.5)		
No	0, (0, 6)			3467 (2880, 3694)		
Professional-technical title^3^		31.098	<0.001		19.224	<0.001
Nurse	1 (0, 7)			3288.5 (2645, 3670)		
Senior nurse	1 (0, 8)			3396 (2743.5, 3686)		
Nurse in charge	1 (0, 10)			3378 (2720, 3679)		
Assistant director nurse or above	1 (0, 12)			3322.5 (2810.75, 3658.75)		
Work seniority (years)^3^		18.109	0.001		20.758	<0.001
<2	0 (0, 7)			3345 (2671.75, 3676.75)		
2–5	1 (0, 9)			3304 (2696.75, 3678)		
5–10	1 (0, 8)			3389 (2714.25, 3685)		
10–20	1 (0, 9)			3399 (2757.5, 3687)		
>20	0 (0, 11)			3309 (2690, 3653.5)		
Shift^2^		−2.820	0.005		−2.786	0.005
Day	1 (0, 8)			3389 (2730, 3683)		
Night	1 (0, 9)			3331.5 (2705, 3674)		
Patient grade ratio^1^						
Special level		0.111	0.912		−4.163	<0.001
First level		2.107	0.045		2.780	0.005
Second level		−0.148	0.882		−3.884	<0.001
Third level		−0.592	0.554		0.747	0.455

*Note*. ^1^linear regression; ^2^one-way ANOVA; ^3^Kruskal–Wallis rank sum test.

**Table 3:** The association among working hours, covariates, and MNC.

		Day shift-6 knots^1^						Night shift-3 knots^2^					
		*B*	SD	Beta	*t*	CI	*p*	*B*	SD	Beta	*t*	CI	*p*
Intercept		−2.980	2.779	—	0.28	−8.43–2.46	0.283	1.070	4.862	—	0.220	−8.46–10.60	0.826

Working hours		0.470	0.357	0.056	1.30	−0.23–1.17	0.192	0.750	0.229	0.161	3.251	0.30–1.19	0.001

Working hours (spline1)		−26.500	8.806	−4.096	−3.01	−43.76–−9.23	0.003	−1.080	0.348	−0.153	−3.102	−1.76–−0.40	0.002

Working hours (spline2)		47.740	15.600	5.139	3.06	17.16–78.31	0.002						

Working hours (spline3)		−80.520	28.110	−1.101	−2.87	−135.62–−25.42	0.004						

Age (years)	26–34	−0.105	0.512	−0.005	−0.205	−1.11–0.90	0.837	0.157	0.625	0.007	0.252	−1.07–1.38	0.801
	35–44	−0.015	0.623	−0.001	−0.023	−1.23–1.21	0.981	−0.333	0.841	−0.011	−0.396	−1.98–1.32	0.692
	≥45	0.277	0.860	0.008	0.322	−1.41–1.96	0.747	−2.284	2.327	−0.019	−0.981	−6.85–2.28	0.326

Overtime work	Yes	2.710	0.308	0.119	8.800	2.10–3.31	<0.001						

Education	Junior college	0.800	1.225	0.027	0.65	−1.60–3.20	0.516	−4.890	4.504	−0.163	−1.085	−13.72–3.94	0.278
	Undergraduate	1.050	1.211	0.037	0.86	−1.33–3.42	0.388	−5.170	4.493	−0.177	−1.150	−13.98–3.64	0.250
	Postgraduate	4.250	1.593	0.041	2.67	1.12–7.37	0.008	−3.770	4.861	−0.031	−0.776	−13.30–5.76	0.438

Professional-technical title	Senior nurse	0.690	0.425	0.033	1.62	−0.14–1.52	0.105	−0.030	0.554	−0.001	−0.055	−1.12–1.06	0.956
	Nurse in charge	1.650	0.498	0.076	3.32	0.68–2.63	0.001	−0.070	0.697	−0.003	−0.099	−1.44–1.30	0.921
	Assistant director nurse or above	2.190	0.757	0.044	2.89	0.70–3.67	0.004	10.830	5.064	0.034	2.138	0.90–20.76	0.033

Work seniority (years)	2–5	−0.360	0.508	−0.012	−0.72	−1.36–0.63	0.473	−0.190	0.609	−0.007	−0.313	−1.39–1.00	0.754
	5–10	−0.670	0.514	−0.030	−1.31	−1.68–0.34	0.191	0.120	0.647	0.005	0.181	−1.15–1.39	0.856
	10–20	−0.760	0.549	−0.034	−1.38	−1.83–0.32	0.169	−0.390	0.714	−0.017	−0.547	−1.79–1.01	0.584
	>20	−0.820	0.657	−0.027	−1.25	−2.11–0.47	0.213	−1.140	1.382	−0.015	−0.823	−3.85–1.57	0.411

N-P ratio		0.010	0.009	0.018	1.39	−0.01–0.03	0.163	0.010	0.013	0.022	0.948	−0.01–0.04	0.343

Patient-care grade (%)	Special-level	−0.177	0.002	−0.001	−0.08	−0.00–0.00	0.939	−0.010	0.005	−0.059	−3.001	−0.02–−0.00	0.003
	First-level	0.449	0.002	0.000	0.02	−0.00–0.00	0.984	0.000	0.003	0.000	0.009	−0.01–0.01	0.993
	Second-level	−0.001	0.001	−0.010	−0.97	−0.00–0.00	0.331	0.010	0.003	0.037	2.269	0.00–0.01	0.023
	Third-level	−0.001	0.003	−0.003	−0.33	−0.01–0.00	0.745	0.010	0.008	0.027	1.687	−0.00–0.03	0.092

Department units	Surgery	0.240	0.269	0.011	0.91	−0.28–0.77	0.362	−1.000	0.423	−0.043	−2.369	−1.83–−0.17	0.018
	Gynecology and obstetrics	−1.570	0.448	−0.038	−3.50	−2.45–−0.69	<0.001	−0.970	0.699	−0.023	−1.388	−2.34–0.40	0.165
	Pediatric	0.020	0.496	0.000	0.03	−0.96–0.99	0.975	−2.020	0.764	−0.043	−2.642	−3.52–−0.52	0.008
	Neonatology	−1.910	1.671	−0.012	−1.14	−5.19–1.36	0.252	−3.200	2.113	−0.024	−1.516	−7.35–0.94	0.130
	Others	−0.250	0.339	−0.008	−0.74	−0.91–0.42	0.462	−0.230	0.561	−0.007	−0.405	−1.33–0.87	0.685
	ICU	−0.480	0.565	−0.009	−0.85	−1.59–0.63	0.398	0.760	0.679	0.020	1.116	−0.57–2.09	0.265
	NICU	−1.740	0.834	−0.022	−2.09	−3.38–−0.11	0.037	0.270	1.032	0.004	0.260	−1.75–2.29	0.795
	PICU	−0.330	1.489	−0.002	−0.22	−3.25–2.59	0.823	−1.800	1.759	−0.016	−1.024	−5.25–1.65	0.306
	Specialist ICU	−0.590	0.668	−0.009	−0.88	−1.89–0.72	0.381	1.950	0.758	0.045	2.568	0.46–3.43	0.010

Labor resource		0.380	0.036	0.142	10.52	0.31–0.45	<0.001	0.520	0.053	0.190	9.706	0.41–0.62	<0.001

Supplies		0.070	0.068	0.018	0.98	−0.07–0.20	0.328	−0.020	0.103	−0.005	−0.200	−0.22–0.18	0.842

Communication		−0.050	0.050	−0.027	−1.09	−0.15–0.04	0.277	0.150	0.071	0.072	2.069	0.01–0.29	0.039

Management		0.270	0.043	0.137	6.33	0.19–0.35	<0.001	0.150	0.063	0.073	2.349	0.02–0.27	0.019

*Note*. ^1^: *F* = 30.77, *p* < 0.001, *R*^2^ = 0.106, adjusted *R*^2^ = 0.103; ^2^: *F* = 14.39 *p* < 0.001, *R*^2^ = 0.104, adjusted *R*^2^ = 0.097.

**Table 4:** The association among working hours, covariates, and NPE.

		Day shift-4 knots^1^						Night shift-4 knots^2^					
		*B*	SD	Beta	*t*	CI	*p*	*B*	SD	Beta	*t*	CI	*p*
Intercept		3164.600	183.000	—	17.294	2805.91–3523.30	<0.001	3631.000	390.300	—	9.305	2866.21–4396.47	<0.001

Working hours		6.620	22.390	0.011	0.295	−37.27–50.50	0.768	−75.410	26.770	−0.225	−2.817	−127.89–−22.93	0.005

Working hours^1^		−310.820	126.000	−0.576	−2.467	−557.83–−63.81	0.014	1388.000	296.500	2.440	4.681	806.52–1969.02	<0.001

Working hours^2^		1699.770	690.000	0.508	2.463	347.23–3052.30	0.014	−3177.000	657.000	−2.214	−4.835	−4464.68–−1888.59	<0.001

Overtime work yes		−229.150	19.850	−0.143	−11.544	−268.06–−190.24	<0.001	−318.100	27.840	−0.196	−11.429	−372.71–−263.57	<0.001

Education	Junior school	−108.810	88.040	−0.052	−1.236	−281.38–63.77	0.217	−122.400	332.100	−0.056	−0.369	−773.56–528.71	0.712
	Undergraduate	16.240	87.050	0.008	0.187	−154.39–186.88	0.852	82.290	331.400	0.039	0.248	−567.36–731.95	0.804
	Postgraduate	38.370	114.500	0.005	0.335	−186.09–262.83	0.738	148.000	358.800	0.017	0.412	−555.58–851.51	0.680

N-P ratio		−3.610	0.650	−0.073	−5.558	−4.88–−2.34	<0.001	1.452	0.938	0.037	1.548	−0.39–3.29	0.122

Professional-technical-title	Senior nurse	38.790	30.560	0.026	1.269	−21.11–98.69	0.204	34.090	40.800	0.023	0.835	−45.91–114.09	0.404
	Nurse in charge	−9.300	35.760	−0.006	−0.260	−79.40–60.80	0.795	63.070	51.360	0.037	1.228	−37.62–163.76	0.219
	Assistant director nurse or above	58.570	54.390	0.017	1.077	−48.06–165.20	0.282	−395.700	373.300	−0.017	−1.060	−1127.70–336.24	0.289

Work seniority (years)	2–5	−13.920	36.510	−0.007	−0.381	−85.48–57.64	0.703	−83.870	44.920	−0.045	−1.867	−171.94–4.19	0.062
	5–10	10.980	36.910	0.007	0.298	−61.38–83.34	0.766	−121.300	47.700	−0.077	−2.542	−214.78–−27.73	0.011
	10–20	37.180	39.460	0.024	0.942	−40.16–114.53	0.346	−84.400	52.650	−0.051	−1.603	−187.62–18.82	0.109
	>20	−4.370	47.190	−0.002	−0.093	−96.88–88.14	0.926	9.107	101.900	0.002	0.089	−190.70–208.91	0.929

Department units	Surgery	47.930	19.300	0.030	2.484	10.10–85.76	0.013	74.150	31.150	0.044	2.380	13.08–135.22	0.017
	Gynecology and obstetrics	23.850	32.220	0.008	0.740	−39.30–87.00	0.459	49.560	51.680	0.016	0.959	−51.75–150.88	0.338
	Pediatric	−56.120	35.630	−0.017	−1.575	−125.96–13.71	0.115	18.630	56.390	0.005	0.330	−91.93–129.19	0.741
	Neonatology	30.780	120.100	0.003	0.256	−204.66–266.23	0.798	155.000	156.100	0.016	0.993	−151.06–461.07	0.321
	Others	52.170	24.360	0.025	2.142	4.42–99.92	0.032	76.760	41.420	0.033	1.853	−4.44–157.96	0.064
	ICU	69.940	40.520	0.020	1.726	−9.49–149.37	0.084	77.760	50.270	0.029	1.547	−20.80–176.31	0.122
	NICU	150.750	59.950	0.027	2.515	33.24–268.26	0.012	158.300	76.540	0.036	2.068	8.23–308.35	0.039
	PICU	345.750	106.900	0.034	3.235	136.23–555.26	0.001	146.300	129.900	0.018	1.126	−108.35–400.91	0.260

Department units	Specialist ICU	135.560	47.900	0.031	2.830	41.66–229.46	0.005	244.600	55.990	0.078	4.368	134.80–354.35	<0.001

Patient-care grade (%)	Special level	−0.710	0.167	−0.050	−4.228	−1.03–−0.38	<0.001	−0.455	0.337	−0.027	−1.349	−1.12–0.21	0.177
	First level	0.840	0.164	0.054	5.087	0.51–1.16	<0.001	0.009	0.217	0.001	0.041	−0.42–0.44	0.967
	Second level	−0.140	0.107	−0.014	−1.309	−0.35–0.07	0.191	−0.942	0.196	−0.080	−4.812	−1.33–−0.56	<0.001
	Third level	−0.090	0.188	−0.005	−0.485	−0.46–0.28	0.627	0.623	0.580	0.017	1.074	−0.51–1.76	0.283

*Note*. ^1^: *F* = 17.9, *p* < 0.001, *R*^2^ = 0.063, adjusted *R*^2^ = 0.059; ^2^: *F* = 9.55, *p* < 0.001, *R*^2^ = 0.067, adjusted *R*^2^ = 0.060.

**Table 5:** Comparison of QNC across groups (*N* = 12703).

Characteristics	*N* (%)				*F*/*τ*b/*Z*	*p*
	Poor	Average	Good	Excellent		
N-P ratio^1^					52.77	<0.001
Departments^2^					0.065	<0.001
Internal medicine	7 (0.17)	486 (11.67)	1908 (45.80)	1765 (42.37)		
Surgery	4 (0.11)	367 (10.11)	1595 (43.95)	1663 (45.83)		
Gynecology and obstetrics	2 (0.23)	67 (7.85)	361 (42.27)	424 (49.65)		
Pediatric	1 (0.15)	85 (12.88)	276 (41.82)	298 (45.15)		
Neonatology	0 (0.00)	2 (3.39)	27 (45.76)	30 (50.85)		
Others	4 (0.23)	166 (9.43)	756 (42.93)	835 (47.42)		
ICU	3 (0.41)	79 (10.85)	330 (45.33)	31 (43.41)		
NICU	0 (0.00)	19 (6.93)	96 (35.04))	159 (58.03)		
PICU	1 (0.15)	4 (4.94)	28 (34.57)	48 (59.26)		
Specialist ICU	0 (0.00)	42 (8.55)	200 (40.73)	249 (50.71)		
Age (years)^2^					−0.032	0.200
≤25	3 (0.16)	170 (9.29)	765 (41.80)	892 (48.74)		
26–34	11 (0.15)	777 (10.39)	3328 (44.52)	3360 (44.94)		
35–44	4 (0.16)	280 (10.92)	1102 (42.96)	1179 (45.96)		
≥45	4 (0.48)	90 (10.82)	382 (45.91)	356 (42.79)		
Gender^3^					−0.611	0.541
Male	3 (1.64)	36 (2.73)	150 (2.69)	172 (2.97)		
Female	19 (86.36)	1281 (97.27)	5427 (97.31))	5615 (97.03)		
Education background					0.014	0.015
Special secondary school	0 (0.00)	6 (7.79)	31 (40.26)	40 (51.95)		
Junior college	4 (0.22)	204 (11.11)	853 (46.46)	775 (42.21)		
Undergraduate	18 (0.17)	1091 (10.23)	4641 (43.50)	4919 (46.11)		
Postgraduate	0 (0.00)	16 (13.22)	52 (42.98)	53 (43.80)		
Overtime work^3^					−23.719	<0.001
Yes	16 (72.73)	688 (52.24)	1930 (34.61)	1219 (21.06)		
No	6 (27.27)	629 (47.76)	3647 (65.39)	4568 (78.94)		
Professional-technical title^2^					−0.036	0.006
Nurse	4 (0.19)	190 (9.20)	872 (42.41)	1000 (48.40)		
Senior nurse	8 (0.14)	617 (10.43)	2616 (44.21)	2676 (45.23)		
Nurse in charge	9 (0.21)	471 (10.94)	1882 (43.73)	1942 (45.12)		
Assistant director nurse or above	1 (0.24)	39 (9.38)	207 (49.76)	169 (40.63)		
Work seniority (years)^2^					−0.022	0.012
<2	2 (0.15)	122 (8.89)	540 (39.36)	708 (51.60)		
2–5	1 (0.05)	203 (10.11)	919 (45.77)	885 (44.07)		
5–10	9 (0.22)	427 (10.45)	1833 (44.84)	1819 (44.50)		
10–20	5 (0.13)	42110.69)	1692 (42.97)	1820 (46.22)		
>20	5 (0.39)	14411.10)	593 (45.72)	555 (42.79)		
Shift^3^					−4.425	<0.001
Day	18 (0.20)	879 (9.95)	3797 (43.00)	4137 (46.85)		
Night	4 (0.10)	438 (11.31)	1780 (45.97)	1650 (42.61)		
Patient grade ratio^1^						
Special level					6.237	<0.001
First level					2.878	0.035
Second level					3.186	0.023
Third level					1.779	0.149

*Note*. ^1^one-way ANOVA; ^2^Kendall tau-b correlation coefficient; ^3^Wilcoxon rank sum test.

**Table 6:** The association among working hours, covariates, and QNC.

		Day shift-4 knots						Night shift-4 knots					
		*B*	SD	*t*	OR	CI	*p*	*B*	SD	*t*	OR	CI	*p*
Poor|average		−7.12	0.51	−14.10	0.000	0.00–0.00	<0.001	−8.249	1.151	−7.166	0.000	0.00–0.00	<0.001

Average|good		−3.07	0.45	−6.84	0.050	0.02–0.11	<0.001	−3.371	1.037	−3.250	0.020	0.01–0.06	<0.001

Good|excellent		−0.63	0.45	−1.42	0.530	0.22–1.28	0.157	−0.905	1.036	−0.874	0.240	0.08–0.68	0.008

Working hours		0.00	0.06	−0.03	1.000	0.88–1.13	0.977	−0.163	0.072	−2.280	0.850	0.74–0.98	0.023

Working hours (spline1)		−0.77	0.34	−2.23	0.460	0.24–0.91	0.026	3.110	0.795	3.914	22.430	4.75–107.20	<0.001

Working hours (spline2)		4.42	1.88	2.35	83.510	2.07–3350.34	0.019	−7.080	1.762	−4.017	0.000	0.00–0.03	<0.001

Overtime work-yes		−0.70	0.05	−13.08	0.500	0.45–0.55	<0.001	−0.893	0.075	−11.883	0.410	0.35–0.47	<0.001

Professional-technical title	Senior nurse	0.01	0.08	0.17	1.010	0.86–1.19	0.862	−0.173	0.104	−1.659	0.840	0.69–1.03	0.097
	Nurse in charge	0.00	0.10	−0.04	1.000	0.83–1.20	0.971	−0.181	0.132	−1.374	0.830	0.64–1.08	0.170
	Assistant director nurse or above	0.02	0.15	0.11	1.020	0.76–1.35	0.916	−3.248	1.094	−2.970	0.040	0.00–0.27	0.003

Work seniority (years)	2–5	−0.24	0.10	−2.37	0.790	0.65–0.96	0.018	−0.239	0.118	−2.019	0.790	0.62–0.99	0.044
	5–10	−0.30	0.10	−2.95	0.740	0.60–0.90	0.003	−0.121	0.126	−0.956	0.890	0.69–1.13	0.339
	10–20	−0.27	0.11	−2.44	0.770	0.62–0.95	0.015	−0.018	0.139	−0.130	0.980	0.75–1.29	0.897
	>20	−0.43	0.13	−3.35	0.650	0.51–0.84	0.001	0.007	0.269	0.026	1.010	0.60–1.71	0.980

N-P ratio		−0.01	0.00	−5.38	0.990	0.99–0.99	<0.001	−0.003	0.003	−1.111	1.000	0.99–1.00	0.267

Department units	Surgery	0.09	0.05	1.66	1.090	0.98–1.21	0.096	0.106	0.082	1.289	1.110	0.95–1.31	0.198
	Gynecology and obstetrics	0.12	0.09	1.33	1.120	0.95–1.33	0.184	0.311	0.139	2.238	1.360	1.04–1.79	0.025
	Pediatric	−0.03	0.10	−0.31	0.970	0.80–1.17	0.754	0.314	0.154	2.039	1.370	1.01–1.85	0.042
	Neonatology	0.35	0.33	1.05	1.420	0.75–2.77	0.296	−0.200	0.404	−0.496	0.820	0.37–1.83	0.620
	Others	0.22	0.07	3.36	1.250	1.10–1.42	0.001	−0.087	0.110	−0.790	0.920	0.74–1.14	0.430
	ICU	0.08	0.11	0.80	1.090	0.88–1.34	0.425	−0.103	0.132	−0.779	0.900	0.70–1.17	0.436
	NICU	0.37	0.17	2.21	1.440	1.05–2.01	0.027	0.379	0.207	1.830	1.460	0.98–2.20	0.067
	PICU	0.47	0.30	1.57	1.600	0.90–2.93	0.116	0.310	0.365	0.849	1.360	0.67–2.84	0.396
	Specialist ICU	0.35	0.13	2.70	1.420	1.10–1.84	0.007	0.076	0.149	0.511	1.080	0.81–1.45	0.610

Patient-care grade (%)	Special level							0.001	0.001	1.312	1.000	1.00–1.00	0.190
	First level							−0.001	0.001	−1.616	1.000	1.00–1.00	0.106
	Second level							−0.001	0.001	−2.088	1.000	1.00–1.00	0.037
	Third level							0.004	0.002	2.301	1.000	1.00–1.01	0.021

## Data Availability

The data used to support the findings of this study are available from the corresponding author upon reasonable request.

## Additional Points

What is already known. (i) Nurses usually work overtime and keep rationing to ensure the continuous nursing care. (ii) Frequent night shifts and working overtime were harmful to the quality of care and nurses' perceptions of the practice environment, resulting in turnover of nurses. (iii) Guidelines by governments and institutions were made to limit the length of work time per shift for nurses. What this paper adds. (i) This analysis provides evidence of a nonliner relationship between work hours and missed nursing care, quality of care, and perception of the nursing practice environment. (ii) This paper proved that working overtime of nurses had a detrimental effect on quality of care and perception of nursing's practice environment whether on the day shift or night shift. (iii) These findings indicated that beneficial working hours are 7–7.75 on the day shift and 12 hours (but no more than 15 hours) on the night shift based on the nurse-reported outcomes.

## References

[1] Ejebu O.-Z., Dall’Ora C., Griffiths P. (2021). Nurses’ experiences and preferences around shift patterns: a scoping review. *PLoS One*.

[2] Baek J., Han K., Choi-Kwon S. (2020). Sleep diary- and actigraphy-derived sleep parameters of 8-hour fast-rotating shift work nurses: a prospective descriptive study. *International Journal of Nursing Studies*.

[3] Larsen A. D., Ropponen A., Hansen J. (2020). Working time characteristics and long-term sickness absence among Danish and Finnish nurses: a register-based study. *International Journal of Nursing Studies*.

[4] Chang Y. S., Wu Y. H., Hsu C. Y., Tang S. H., Yang L. L., Su S. F. (2011). Impairment of perceptual and motor abilities at the end of a night shift is greater in nurses working fast rotating shifts. *Sleep Medicine*.

[5] Korea Health Industry Development Institute (2014). *Survey on Nurses’ Activity Status*.

[6] Baillie L., Thomas N. (2019). Changing from 12-hr to 8-hr day shifts: a qualitative exploration of effects on organising nursing care and staffing. *Journal of Clinical Nursing*.

[7] Dall’Ora C., Griffiths P., Emmanuel T., Rafferty A. M., Ewings S. (2020). 12-hr shifts in nursing: do they remove unproductive time and information loss or do they reduce education and discussion opportunities for nurses? A cross-sectional study in 12 European countries. *Journal of Clinical Nursing*.

[8] Min A., Min H., Hong H. C. (2019). Work schedule characteristics and fatigue among rotating shift nurses in hospital setting: an integrative review. *Journal of Nursing Management*.

[9] Shah M. K., Gandrakota N., Cimiotti J. P., Ghose N., Moore M., Ali M. K. (2021a). Prevalence of and factors associated with nurse burnout in the US. *JAMA Network Open*.

[10] James L., Elkins-Brown N., Wilson M. (2021). The effects of three consecutive 12-hour shifts on cognition, sleepiness, and domains of nursing performance in day and night shift nurses: a quasi-experimental study. *International Journal of Nursing Studies*.

[11] Dall’Ora C., Ball J., Redfern O. (2019). Are long nursing shifts on hospital wards associated with sickness absence? A longitudinal retrospective observational study. *Journal of Nursing Management*.

[12] Rodríguez-García M. C., Márquez-Hernández V. V., Belmonte-García T., Gutiérrez-Puertas L., Granados-Gámez G. (2020). Original research: how Magnet hospital status affects nurses, patients, and organizations: a systematic review. *AJN, American Journal of Nursing*.

[13] Min A., Yoon Y. S., Hong H. C., Kim Y. M. (2020). Association between nurses’ breaks, missed nursing care and patient safety in Korean hospitals. *Journal of Nursing Management*.

[14] Stewart N. H., Arora V. M. (2019). The impact of sleep and circadian disorders on physician burnout. *Chest*.

[15] Griffiths P., Dall’Ora C., Simon M. (2014). Nurses’ shift length and overtime working in 12 European countries: the association with perceived quality of care and patient safety. *Medical Care*.

[16] Ball J., Day T., Murrells T. (2017). Cross-sectional examination of the association between shift length and hospital nurses job satisfaction and nurse reported quality measures. *BMC Nursing*.

[17] D’Sa V. M., Ploeg J., Fisher A., Akhtar-Danesh N., Peachey G. (2018). Potential dangers of nursing overtime in critical care. *Canadian Journal of Nursing Leadership*.

[18] Shin S., Lee I., Kim J., Bae S.-H. (2019). Work-related characteristics and sleep quality of nurses in comprehensive nursing care units of small-medium sized hospitals. *J Korean Acad Fundam Nurs*.

[19] Koy V., Yunibhand J., Angsuroch Y., Torale S. (2017). Development and psychometric testing of the Cambodian nursing care quality scale. *Pacific Rim international journal of nursing research*.

[20] Institute of Medicine Committee on the Work Environment for N., Patient S. (2004). *Keeping Patients Safe: Transforming the Work Environment of Nurses*.

[21] Griffiths P., Dall’Ora C., Sinden N., Jones J. (2019). Association between 12-hr shifts and nursing resource use in an acute hospital: longitudinal study. *Journal of Nursing Management*.

[22] Saville C., Dall’Ora C., Griffiths P. (2020). The association between 12-hour shifts and nurses-in-charge’s perceptions of missed care and staffing adequacy: a retrospective cross-sectional observational study. *International Journal of Nursing Studies*.

[23] National Health Commission of the Poeple’s Republic of China (2011). *Circular of the Ministry of Health on Printing and Distributing Interim Measures for Hospital Evaluation*.

[24] Aiken L. H., Sloane D. M., Bruyneel L., Van den Heede K., Sermeus W. (2013). Nurses’ reports of working conditions and hospital quality of care in 12 countries in Europe. *International Journal of Nursing Studies*.

[25] Cho E., Lee N. J., Kim E. Y. (2016). Nurse staffing level and overtime associated with patient safety, quality of care, and care left undone in hospitals: a cross-sectional study. *International Journal of Nursing Studies*.

[26] McHugh M. D., Stimpfel A. W. (2012). Nurse reported quality of care: a measure of hospital quality. *Research in Nursing & Health*.

[27] Kalisch B. J., Williams R. A. (2009). Development and psychometric testing of a tool to measure missed nursing care. *The Journal of Nursing Administration: The Journal of Nursing Administration*.

[28] Si F. (2019). *Validation of the Chinese Version of Missed Nursing Care Scale and its Application in Nurses and Patients in Hospita*.

[29] Haiyan Z., Zhijun W., Junqing L., Wenhan S., Weiyan J., Li Y. (2019). Revision of the nursing practice environment scale and evaluation of its reliability and validity. *Chinese Nursing Management*.

[30] National Health Commission of People’s republic of China (2013). *Nursing Classfication*.

[31] Lusa L., Ahlin Č. (2020). Restricted cubic splines for modelling periodic data. *PLoS One*.

[32] Harrell F. E. (2001). *Regression Modeling Strategies with Applications to Linear Models, Logistic Regression, and Survival Analysis*.

[33] Steyerberg E. W., Harrell F. R. A. N. K. E. (2016). *Regression Modeling Strategies: With Applications, to Linear Models, Logistic and Ordinal Regression, and Survival Analysis*.

[34] Wu Y., Zheng J., Liu K. (2018). The associations of occupational hazards and injuries with work environments and overtime for nurses in China. *Research in Nursing and Health*.

[35] Ropponen A., Koskinen A., Puttonen S., Härmä M. (2020). A case-crossover study of age group differences in objective working-hour characteristics and short sickness absence. *Journal of Nursing Management*.

[36] Jawahir S., Mohamad Anuar N. N., Sheikh Abdullah S. F., Silvernayagam S., Tan E. H. (2021a). Perception of nurses on the practice environment: experience from Malaysia. *Medical Journal of Malaysia*.

[37] Knupp A. M., Patterson E. S., Ford J. L., Zurmehly J., Patrick T. (2018). Associations among nurse fatigue, individual nurse factors, and aspects of the nursing practice environment. *The Journal of Nursing Administration: The Journal of Nursing Administration*.

[38] Bae S.-H., Pen M., Sinn C. (2021). Work hours and overtime of nurses working in Cambodian hospitals. *International Nursing Review*.

[39] American Nurses Association (2014). *Addressing Nurse Fatigue to Promote Safety and Health: Joint Responsibilities of Registered Nurses and Employers to Reduce Risks*.

[40] National Health Commission of the People’s Republic of China (2016). *National Nursing Development Plan (2016—2020)*.

[41] Health Commission of Hubei Province (2020). *Notice of the Provincial Health Commission on Strengthening Health Education and Health Promotion in Medical Institutions*.

[42] West S., Rudge T., Mapedzahama V. (2016). Conceptualizing nurses’ night work: an inductive content analysis. *Journal of Advanced Nursing (China)*.

[43] Xu R., Hu K. L., Yin S. Y., Wang Y., Wang H. (2015). A study of nurses’ participation in public activities and sleep demand after night shift. *Journal of Nursing*.

[44] Cho S. H., Lee J. Y., You S. J., Song K. J., Hong K. J. (2020). Nurse staffing, nurses prioritization, missed care, quality of nursing care, and nurse outcomes. *International Journal of Nursing Practice*.

[45] Bragadóttir H., Kalisch B. J., Tryggvadóttir G. B. (2017). Correlates and predictors of missed nursing care in hospitals. *Journal of Clinical Nursing*.

[46] Chaboyer W., Harbeck E., Lee B.-O., Grealish L. (2021). Missed nursing care: an overview of reviews. *The Kaohsiung Journal of Medical Sciences*.

[47] Jones T. L., Hamilton P., Murry N. (2015). Unfinished nursing care, missed care, and implicitly rationed care: state of the science review. *International Journal of Nursing Studies*.

[48] Mandal L., Seethalakshmi A., Rajendrababu A. (2020). Rationing of nursing care, a deviation from holistic nursing: a systematic review. *Nursing Philosophy: An International Journal for Healthcare Professionals*.

[49] Mitello L., Marucci A. R., Salvatore S., Sii Onesto A., Baglio G., Latina R. (2021). Predictors of nurses’ attitudes and knowledge towards pain management in Italy. A cross-sectional study in the hospital settings. *Applied Nursing Research*.

[50] Gómez-García T., Ruzafa-Martínez M., Fuentelsaz-Gallego C. (2016). Nurses’ sleep quality, work environment and quality of care in the Spanish National Health System: observational study among different shifts. *BMJ Open*.

[51] Jawahir S., Mohamad Anuar N. N., Sheikh Abdullah S. F., Silvernayagam S., Tan E. H. (2021b). Perception of nurses on the practice environment: experience from Malaysia. *Medical Journal of Malaysia*.

[52] Wong H., Wong M. C. S., Wong S. Y. S., Lee A. (2010). The association between shift duty and abnormal eating behavior among nurses working in a major hospital: a cross-sectional study. *International Journal of Nursing Studies*.

[53] Gensimore M. M., Maduro R. S., Morgan M. K., McGee G. W., Zimbro K. S. (2020). The effect of nurse practice environment on retention and quality of care via burnout, work characteristics, and resilience: a moderated mediation model. *The Journal of Nursing Administration: The Journal of Nursing Administration*.

[54] Chen L., Luo C., Liu S. (2019). Excessive daytime sleepiness in general hospital nurses: prevalence, correlates, and its association with adverse events. *Sleep and Breathing*.

[55] Suter J., Kowalski T., Anaya-Montes M., Chalkley M., Jacobs R., Rodriguez-Santana I. (2020). The impact of moving to a 12h shift pattern on employee wellbeing: a qualitative study in an acute mental health setting. *International Journal of Nursing Studies*.

[56] Caruso C. C., Baldwin C. M., Berger A. (2019). Policy brief: nurse fatigue, sleep, and health, and ensuring patient and public safety. *Nursing Outlook*.

[57] Hopcia K., Dennerlein J. T., Hashimoto D., Orechia T., Sorensen G. (2012). Occupational injuries for consecutive and cumulative shifts among hospital registered nurses and patient care associates: a case-control study. *Workplace Health and Safety*.

